# Unraveling the Use of Disinformation Hashtags by Social Bots During the COVID-19 Pandemic: Social Networks Analysis

**DOI:** 10.2196/50021

**Published:** 2025-01-09

**Authors:** Victor Suarez-Lledo, Esther Ortega-Martin, Jesus Carretero-Bravo, Begoña Ramos-Fiol, Javier Alvarez-Galvez

**Affiliations:** 1 Computational Social Science DataLab University Institute of Research for Sustainable Social Development (INDESS) University of Cadiz Jerez de la Frontera Spain; 2 Department of Sociology University of Granada Granada Spain; 3 Department of General Economy (Sociology Area) Faculty of Nursing and Physiotherapy University of Cadiz Cadiz Spain; 4 Department of Quantitative Methods Universidad Loyola Andalucía Seville Spain

**Keywords:** social media, misinformation, COVID-19, bot, hashtags, disinformation, network analysis, community detection, dissemination, decision-making, social bot, infodemics, tweets, social media network

## Abstract

**Background:**

During the COVID-19 pandemic, social media platforms have been a venue for the exchange of messages, including those related to fake news. There are also accounts programmed to disseminate and amplify specific messages, which can affect individual decision-making and present new challenges for public health.

**Objective:**

This study aimed to analyze how social bots use hashtags compared to human users on topics related to misinformation during the outbreak of the COVID-19 pandemic.

**Methods:**

We selected posts on specific topics related to infodemics such as vaccines, hydroxychloroquine, military, conspiracy, laboratory, Bill Gates, 5G, and UV. We built a network based on the co-occurrence of hashtags and classified the posts based on their source. Using network analysis and community detection algorithms, we identified hashtags that tend to appear together in messages. For each topic, we extracted the most relevant subtopic communities, which are groups of interconnected hashtags.

**Results:**

The distribution of bots and nonbots in each of these communities was uneven, with some sets of hashtags being more common among accounts classified as bots or nonbots. Hashtags related to the Trump and QAnon social movements were common among bots, and specific hashtags with anti-Asian sentiments were also identified. In the subcommunities most populated by bots in the case of vaccines, the group of hashtags including #billgates, #pandemic, and #china was among the most common.

**Conclusions:**

The use of certain hashtags varies depending on the source, and some hashtags are used for different purposes. Understanding these patterns may help address the spread of health misinformation on social media networks.

## Introduction

From the swine influenza (H1N1) pandemic in 2009 to the subsequent outbreak of the H7N9 virus, also known as bird flu, in 2013, Twitter (subsequently rebranded as X) has increasingly become a popular platform for sharing health information [1,2]. Using posts, users can express their thoughts and opinions on many health topics. That is why specific interaction tasks have attracted the attention of researchers. This research can inform public policy by encouraging governments and health care professionals to allocate necessary resources, act, and plan accordingly [3,4]. These social media platforms have played a crucial role in providing information to the public during the COVID-19 pandemic. However, there was an increase in low-quality information, as well as the infodemic phenomenon. The infodemic, defined as an excess of information that makes it difficult for people to find reliable sources [5], can have harmful consequences [6].

The COVID-19 pandemic triggered mandatory lockdowns, social distancing, quarantines, and SARS-CoV-2–protective measures that would give rise to all sorts of opinions and behaviors [7]. During the COVID-19 pandemic, mandatory lockouts drastically altered people’s daily routines (work, travel, and leisure activities) to levels never before experienced by the populations of the different countries affected by the new disease [8]. The state of uncertainty in the face of an invisible threat would transform previously normal situations into situations of risk. Direct social interaction with people outside the nuclear family, attending a concert, meeting for dinner with friends and family, shaking hands with someone, and even hugging or kissing became exceptional situations during the most uncertain periods of the pandemic—situations that, as has been observed retrospectively, would have a significant impact on the mental health of the population [9]. Likewise, the health crisis gave rise to the infodemic that, through social media platforms, opened the door to fake news, misconceptions, hoaxes, and anecdotal evidence about the origin of the pandemic, the social agents to blame for the situation, and the possible measures to be taken at a time of maximum uncertainty [10].

To understand how during the new context of health emergency misinformation spreads on these platforms, studies analyzed different elements, including the quality of information sources through URL analysis; identification of topics that generate misinformation; and analysis of online communities that spread misinformation, such as the antivaccine movement [11-14]. Others focused on the use of hashtags to describe the organization of the debate around the COVID-19–related topics. Researchers examined the frequency of use and the topic analysis of hashtags, and emphasized their main role in certain conversations [15,16]. By analyzing specific hashtags, studies also demonstrated how antivaccine communities, the proliferation of racist sentiments, or the spread of conspiracy theories are articulated on social media [17-19]. Some studies paid particular attention to how hashtags were used or combined in online conversations about the COVID-19 pandemic, using clustering techniques to describe the themes and combining hashtags with semantic text analysis and natural language processing (NLP) methods to improve topic modeling [20-22]. In addition, social network analysis (SNA) became useful to examine the co-occurrence of hashtags [23]. These studies demonstrate how the combination of different approach is useful to analyze online conversations more thoroughly.

Recently, the role of social bots has contributed to the spread of misinformation on social media platforms in various ways [24]. This issue garnered more attention as fake news and misinformation were significant factors during the COVID-19 pandemic. In this sense, some studies analyzed the role of bots regarding the spread of misinformation in general, while others have focused specifically on topics such as vaccines, conspiracy theories, hate speech, or reactions to other political actions [25-31]. However, a small amount of research compared the behavior of bots and humans [32,33].

To better understand the influence of bots on social media conversations, a previous study used topic modeling to segment the Twitter conversation and compare differences between accounts [34]. Nevertheless, the analysis did not focus on the usage of hashtags, which is the primary focus of this study. We aim to identify patterns and trends in hashtag usage to describe how bots and nonbots differ in their use of hashtags.

Only a few studies analyzed how social media bots use hashtags compared to humans. Most studies in this field examine specific hashtags [17-19,35-37]. To address this knowledge gap, we explore how social bots use hashtags specifically in connection with certain infodemic topics, issues that contribute to the generation or spread of fake news, misinformation, or discriminatory narratives. By analyzing how frequently hashtags co-occur, we aim to understand how they appear in the conversation and how they are combined. Besides, we also considered the context in which hashtags are used. They can be used ironically or convey disagreement. Our goal is to address three key questions: (1) What are the most common hashtag co-occurrences? (2) What are the differences in hashtag usage between bots and nonbots? and (3) Do bots and nonbots use certain hashtags in different ways?

## Methods

### Data Collection

Data collection for this study took place from March 16 to June 15, 2020, using the Twitter Streaming application programming interface (API). The hashtags #covid_19, #covid19, #covid, and #coronavirus were used to capture conversations about the first wave of COVID-19 pandemic, and only English-language posts were selected.

Based on previous research, we created a list of topics that were commonly associated with fake news or misinformation. This list includes ozone, laboratory, 5G, conspiracy, Bill Gates, milk, military, and UV. Vaccines were also identified as a controversial topic in multiple studies, so we added them to the list [38-40].

### Ethical Considerations

The present study was approved by the Ethics Committee of the University of Cadiz (005_2024).

### Bot Classification

To identify whether accounts on Twitter were bots or not, we used Botometer by OsoMe (formerly known as BotOrNot) [41]. This publicly available application uses over a thousand criteria to determine how closely a Twitter account matches the typical characteristics of social bots.

To create a binary classification (bot or nonbot) and prioritize identifying true positives over true negatives, we set a threshold value of 0.8 [34]. Using this threshold, we classified approximately 14.8% of the accounts as bots, which is in line with the findings of other research that found bot levels to be between 9% and 15% of the total number of Twitter accounts [42].

Botometer also provides rankings for 6 main types of bots, including echo-chamber, fake follower, financial, self-declared, spammer, and others, in addition to the overall likelihood of being a bot. In this study, we focused on analyzing the behavior of social bot accounts, particularly those that were not identified as automated accounts. These types of accounts are often associated with press agencies, companies, newspapers, or journals, and their primary purpose is to automatically publish information about a specific topic. These accounts may indicate that they are automated, for example, by including the word “bot” in their screen name or being identified as bots on Botwiki [41]. Therefore, we chose to exclude self-declared bots from our analysis due to their different characteristics compared with other social bots [41].

For this study, we classified accounts as nonbots if their probability of being a bot was less than 0.8, as self-declared bots if their probability of being a self-declared bot was greater than 0.8, and as bots if their probability of being a bot was greater than 0.8 and their probability of being a self-declared bot was less than 0.8. We then filtered out self-declared bots and considered both bots and nonbots for analysis.

### Network Analysis

To identify patterns in the usage of hashtags, we applied network analysis. We constructed a network by analyzing the co-occurrence of hashtags in posts and comparing the use of hashtags by bots and nonbots. In the network, hashtags were represented as nodes, and they were connected if they appeared in the same post. The weight of the connection between 2 hashtags was determined by the number of times they co-occurred.

**Figure 1:**
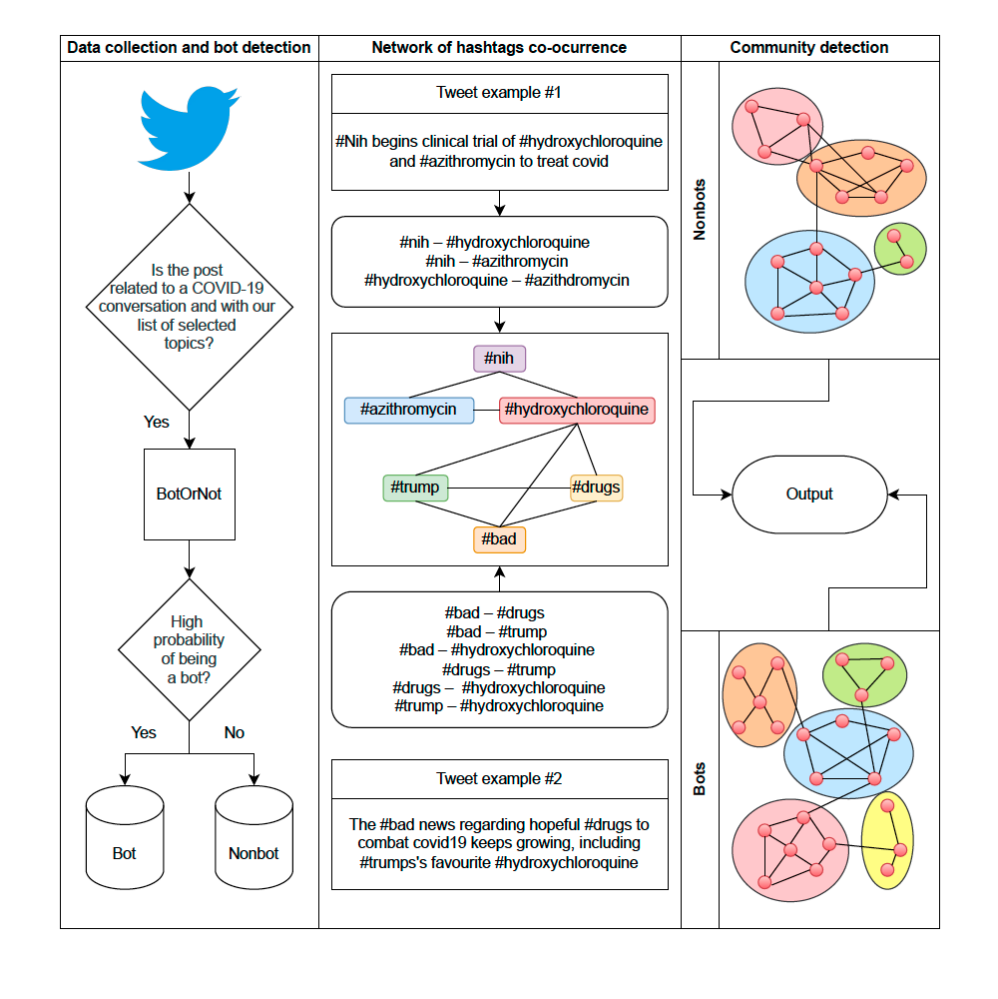
Flowchart from data collection to analysis.

We also calculated various metrics of connection, distribution, and segmentation of the hashtag network. We used the PageRank algorithm to identify the most important nodes in the network and the degree value, which represents the number of connections each hashtag has [43]. We also used the betweenness metric, which measures centrality [44]. In addition, we used the Louvain algorithm to detect the most important communities in the network. This algorithm maximizes a modularity score for each community, where the modularity measures the quality of the assignment of nodes to communities. This allowed us to identify hashtags that often co-occur together. We computed each metric separately considering whether the hashtags appear in posts posted by a bot or a nonbot. Figure 1 contains a flow diagram for the entire process.

In the following section, we first present the results for the entire network. In the following subsections, 1 for each topic, we segment the overall network of hashtag co-occurrences by extracting posts that specifically mention words related to each topic. For example, the network for vaccines will show the co-occurrences of all hashtags that appeared in posts about vaccines.

## Results

### Overview

**Figure 2:**
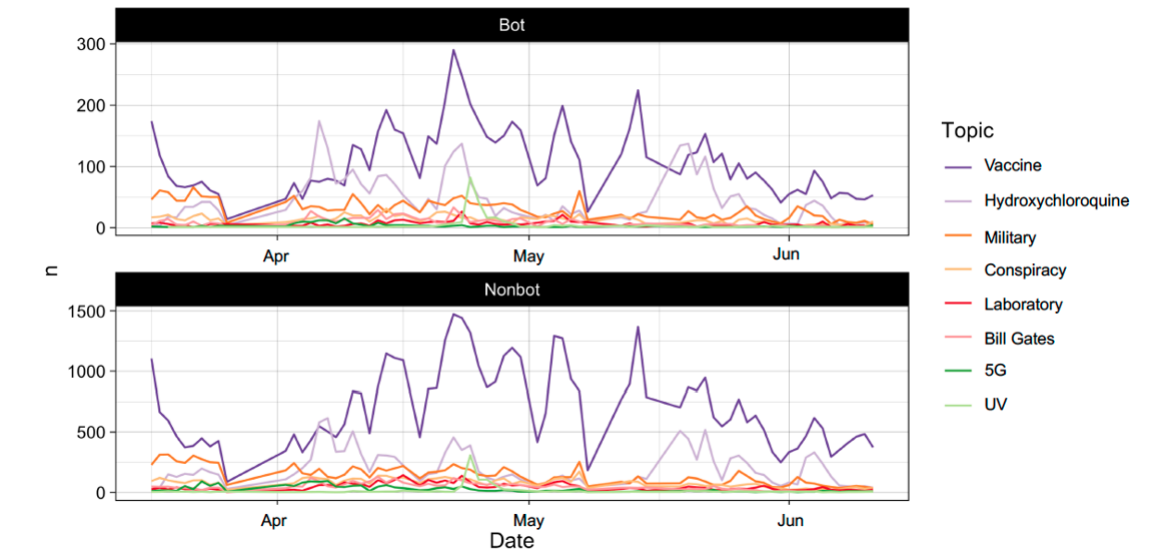
Bot and nonbot distribution by topic and date.

In total, we extracted around 107,173 posts from March to July 2020 that were related to the topics on our list. Most of these posts were about vaccines (59,090/107,173, 55.1%), hydroxychloroquine (17,731/107,173, 16.5%), or the military (12,548/107,173, 11.5%). Out of all the accounts analyzed, 85.2% (91,311/107,173) were identified with a low likely of being bots, that is, nonbots. Approximately 14.8% (15,862/107,173) of the posts were classified as likely being from bot accounts. As shown in Figure 2, the number of posts related to vaccines was consistently higher throughout the period, except for 2 specific moments. The first of these coincides with a message from US President Donald Trump recommending the use of hydroxychloroquine, an unproven drug. The second date also coincides with a message from Trump suggesting the injection of disinfectant to beat COVID-19 pandemic.

**Figure 3:**
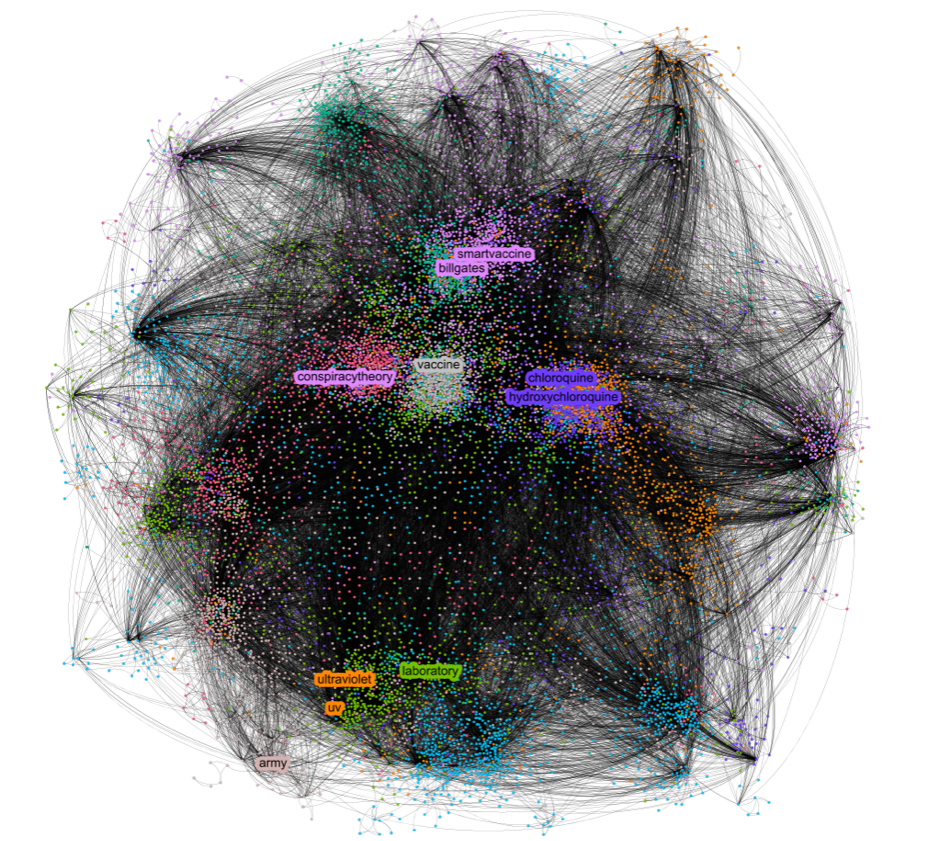
Hashtag network.

We created a graph of the full network of hashtags. For clarity, we selected a random sample from the entire collection of posts and depicted it in Figure 3. We also applied color to the Louvain communities and highlighted some hashtags that represent the topics analyzed in the study. This process is like the one we used for each topic in the list.

In Table 1, we present statistics for the overall hashtags network to provide a broad overview. As mentioned earlier, we calculated the metrics separately for each type of account. There are some differences in the most used hashtags between the 2 groups. For example, hashtags such as #Trump, #China, and #BillGates appear in both groups. However, the hashtag #vaccineswork is one of the most used by nonbots, while the hashtag #lka (which is the country code for Sri Lanka) is more frequently used by bots.

**Table 1:** Most common co-occurrences by bot and nonbot.

Hashtags		Posts, n (%)
**Bots (n=3459)**		
	#chloroquine - #hydroxychloroquine	537 (15.52)
	#hydroxychloroquine - #trump	490 (14.17)
	#africaisnotalaboratory - #changeyourworld	437 (12.63)
	#azithromycin - #hydroxychloroquine	345 (9.97)
	#coronavirushoax - #prisonearth	280 (8.09)
	#digitalvirus - #policestate	280 (8.09)
	#digitalvirus - #prisonearth	280 (8.09)
	#policestate - #prisonearth	280 (8.09)
	#coronaviruslockdown - #lockdownextension	267 (7.72)
	#changeyourworld - #coronacrisisuk	263 (7.6)
**Nonbots (n=665)**		
	#hydroxychloroquine - #trump	133 (20)
	#climatechange - #sustainability	106 (15.94)
	#lka - #srilanka	86 (12.93)
	#chloroquine - #hydroxychloroquine	84 (12.63)
	#azithromycin - #hydroxychloroquine	72 (10.83)
	#kag - #maga	53 (7.97)
	#pandemic - #vaccine	35 (5.26)
	#billgates - #vaccines	33 (4.96)
	#kag - #qanon	33 (4.96)
	#china - #vaccine	30 (4.51)

There are also some similarities in the co-occurrence of hashtags between the 2 groups. For example, hashtags #hydroxychloroquine and #trump appear in the same posts with higher frequency in both cases, at 14.17% (490/3459) and 20% (133/665), respectively. However, other hashtag pairs such as #kag-#maga, #billgates-#vaccines, or #kag-#qanon are common among bots. “KAG” stands for “Keep America Great,” which was President Trump’s campaign slogan in 2020, while “MAGA” stands for “Make America Great Again,” which was his campaign slogan in 2016. Both slogans have been associated with American nationalism, and the hashtag #MAGA has sometimes been used by white supremacist groups and Trump supporters.

On the other hand, nonbots tend to use other hashtag pairs such as #coronavirushoax-#prisionearth, #digitalvirus-#policestate, and #digitalvirus-#prisionearth. These hashtags, especially “#prisionearth,” were often used ironically to mock false rumors or exaggerations that were circulated online.

### Vaccines

The most common co-occurrent hashtags used by nonbots regarding vaccines are #uk-#usa, #research-#science, #vaccineswork-#worldimmunizationweek. However, the most common hashtags in those posts posted by bots are #trump-#votebluetosaveamerica, #healthcare-#ppe, or even #healthcare-#ventilators. In addition, these last mentioned are exclusive of bots. That is, they only co-occur in posts from accounts classified as bots. Besides, it is worth mentioning that #billgates, along with #pandemic or #china, are the hashtags with the highest degree of connections, as seen in Table 2.

**Table 2:** Most important hashtags by topic.

Hashtags		Degree		PageRank		Betweenness
**Vaccine**						
	billgates	44	0.025		22,728	
	pandemic	39	0.019		26,196	
	china	35	0.019		12,380	
	usa	30	0.013		7,375	
	vaccineswork	28	0.019		8,833	
	trump	28	0.015		15,704	
	stayhome	22	0.011		4,583	
	uk	21	0.010		2,703	
	science	21	0.011		5,048	
	france	19	0.008		2,064	
**Military**						
	trump	34	0.042		8,032	
	china	27	0.030		3,733	
	usa	22	0.026		5,561	
	italy	16	0.023		4,219	
	us	16	0.019		1,667	
	iran	15	0.020		1,938	
	russia	11	0.015		1,353	
	maga	10	0.012		620	
	wuhan	10	0.012		497	
	breaking	9	0.012		2,372	
**Laboratory**						
	wuhan	36	0.045		8,422	
	laboratory	26	0.033		11,660	
	africaisnotalaboratory	21	0.041		4,641	
	china	20	0.023		3,470	
	staysafe	11	0.017		7,566	
	stayhome	10	0.013		9,242	
	us	8	0.009		476	
	pandemic	8	0.009		8,614	
	coronaviruslockdown	7	0.011		1,676	
	healthcare	7	0.009		1,331	
**5G**						
	china	42	0.020		31,413	
	pandemic	27	0.012		25,136	
	wuhan	19	0.009		13,463	
	iot	18	0.008		11,045	
	qanon	17	0.008		6,437	
	bigdata	17	0.007		7,446	
	technology	17	0.008		8,731	
	ai	14	0.007		4,819	
	tech	14	0.006		4,455	
	fakenews	14	0.007		8,353	
**Hydroxychloroquine**						
	trump	54	0.074		10,106	
	chloroquine	20	0.028		2,538	
	coronaviruspandemic	15	0.020		1,515	
	kag	14	0.017		897	
	maga	13	0.017		2,197	
	coronavirusoutbreak	12	0.016		1,089	
	india	12	0.017		855	
	hcq	12	0.020		1,468	
	usa	12	0.015		2,095	
	gop	11	0.014		636	
**Conspiracy**						
	conspiracy	35	0.084		1,872	
	conspiracytheory	25	0.054		2,111	
	conspiracytheories	16	0.037		686	
	pandemic	16	0.033		878	
	china	15	0.032		785	
	trump	12	0.030		732	
	disinformation	10	0.022		77	
	fakenews	10	0.023		321	
	usa	10	0.024		778	
	us	9	0.020		213	
**Bill Gates**						
	billgates	68	0.056		17,637	
	qanon	29	0.023		4,043	
	pandemic	27	0.024		7,341	
	maga	23	0.017		1,650	
	vaccines	19	0.016		5,232	
	stopbillgates	15	0.011		862	
	kag	13	0.009		104	
	trump	13	0.011		1,049	
	microsoft	13	0.010		1,978	
	usa	13	0.010		1,173	
**UV**						
	ai	14	0.041		839	
	trump	11	0.044		1,427	
	health	8	0.025		491	
	innovation	8	0.024		171	
	pandemic	8	0.029		428	
	uvlight	8	0.028		1,617	
	robots	7	0.023		754	
	artificialintelligence	6	0.018		112	
	lysol	5	0.018		122	
	machinelearning	5	0.016		255	

The algorithm extracted 5 different communities (Multimedia Appendix 1). We found significant differences in the hashtags that made up the Louvain communities. The first community contains hashtags related to news (#breaking, #usnews, and #breakingnews); countries (#canada, #france, #japan, #spain, and #africa); and others related to fake news like #wuhanvirus, #ccpvirus, #bioweapon, #hiddenhand, #psychopaths, #chinaisassho, and #madeinchina. This community is the most populated by bots, and the difference between bots and nonbots is the highest.

The second community contains hashtags related to famous people (#billgates, #anthonyfauci, and #georgesoros). These include people like Bill Gates and Anthony Fauci who played a leading role by holding provaccine positions. As in the previous case, we also found some hashtags related to fake news or conspiracy theories such as #billgatesisevil, #billgatesvaccine, #vaccinemafia, or #newworldorder. In this community, the quantity of nonbots is slightly higher than the number of bots.

On the other hand, the number of bots is also higher in the third community. In this case, the hashtags mention politics, such as #trump, #biden, and #borisjohnson. In addition, there were also some hashtags related to measures to curb the pandemic, such as #stayhome, #socialdistancing, or #lockdown. Only a few infodemic-related hashtags were found: #methanemouth, #pussygrabber, or #bananarepublic. The number of nonbots is higher in the other 2 communities. The fourth and fifth communities contain hashtags related to research and vaccines (#research, #health, and #medicine) or diseases and public health campaigns (#vaccineswork, #measles, #endpolio, and #healthforall), respectively. In particular, #vaccineswork is a hashtag used by health institutions such as the World Health Organization. Conversations on these hashtags were related to second waves and the importance of vaccines to fight against the COVID-19 pandemic.

### Hydroxychloroquine

Hashtags related to Trump and the Republican movement were common in the case of hydroxychloroquine. These hashtags, such as #kag, #maga, #gop, #qanon, and #tcot, were more common in bot posts. Although #trump also appears in the case of nonbots, there were other hashtags related to news: #breaking-#breakingnew and #chinavirus-#wuhanvirus. Consequently, #trump has the highest degree of connection and the one with the highest betweenness. This hashtag, along with #chloroquine or #coronaviruspandemic, is the hashtag with the highest number of connections. There is a big difference between the first and the rest of the hashtags shown in Table 2. This difference indicates the leading role that #trump plays in the conversation about hydroxychloroquine.

We identified 8 different communities (Multimedia Appendix 1). Regarding the composition of the communities, it is worth mentioning the difference between the 2 most important ones. On the one hand, the first contains hashtags related to drugs, vaccines, or the pharmaceutical industry: #azithromycin, #biotech, #chloroquine, #lupus, #malaria, #cdc, or #hcq. In the same line, in the fourth community, the predominance of nonbots is noticeable. This time the hashtags mention countries (#uk, #us, #coronavirusuk, #france, #italy, and #germany), news (#worldnews and #usnews), TV series (#greysanatomy and #littlefireseverywhere), and supporting hashtags (#inthistogether).

On the other hand, in the second community, most of the hashtags are related to Trump or social movements related to him (#trump, #gop, #maga, and #donaldtrump). Nonetheless, some are against him (#notaleader, #worstpresidentinhistory, and #putinpuppet). In addition, the number of bots is higher than the number of nonbots, contrary to what happens in the first one.

### Military

In this case, hashtags are related to specific countries that were mentioned during the pandemic. For nonbots, those most mentioned are #china-#us, #italy-#russia, and #lka-#srilanka. The latter is the most common among bots, followed in fourth place by #italy-#russia. Among the sets that do not mention countries, we find hashtags related to Trump (#gop-#trump, #kag-#maga, and #kag-#qanon).

These hashtags have similarities to those of hydroxychloroquine. The bots’ unique hashtags are related to the Trump movement or Republican movements (#gop, #kag, and #qanon). In addition, #trump has the highest degree of connectivity and betweenness. This situation is also present in the communities (Multimedia Appendix 1). The first community detected contains hashtags related to Trump, and the second is related to military and veterans (#usmc, #veterans, or #usairforce). In both cases, these relationships take place in posts posted by bots.

### Conspiracy

In this group, we found some hashtags related to conspiracy theories (or misinformation) and others related to countries. Regarding bots, the most common hashtags are #fakenews-#technology, #conspiracytheories-#socialmedia, and #donthecon-#trumplies. In line with this, for the nonbots, the most common hashtags are #conspiracytheory-#woke. The hashtags used only by bots are also related to racism (#racism-#sinophobia) or the economic system (#capitalismfails-#socialismworks).

Of the 6 most prominent communities (Multimedia Appendix 1), 3 of them have only nonbots. Topics in these communities are about minority groups (#blackpeople, #lgbt, and #amerikkka), about Trump (#maga, #bananarepublic, and #qanon), and about the pandemic (#coronavirusoutbreak, #coronaviruspandemic, and #pandemictech). Of the other 3, in the first one, the number of nonbots is slightly higher than the number of bots. Some of the hashtags have to do with conspiracy theories (#conspiracytheory, #disinformation, and #propaganda), media (#qanonnfoxnews, #propaganda, and #fakenews), and others in a derogatory tone (#covidiot, #plandemic, and #plandemicdocumentary). On the other hand, in the second and fifth communities, the numbers of bots are higher. In this case, the most common hashtags are related to countries (#china, #us, and #iran), Iran specifically (#irancovidtruth and #iranregimechange), or against right-wing political parties (#rightwingignorance).

### Laboratory

In this case, there are apparent differences in the geographical areas of the most used hashtags. On the one hand, nonbots mostly use #africaisnotalaboratory, while bots use #srilanka and #lka (country code for Sri Lanka). The hashtag #indiafightscorona is also common for bots. The hashtags #china-#wuhan are very common in both cases. This explains why #wuhan is the hashtag with the highest PageRank value and the highest degree of connection (Table 2), followed by #laboratory in second place and #africaisnotalaboratory in third place.

The differences between hashtags and the type of account that wrote the message were very clear in this case. On the one hand, in the first and fourth communities, the presence of bots is higher than nonbots (Multimedia Appendix 1). The first is focused on China, with some examples such as #ccpvirus, #chinamustexplain, or #chinaliedpeopedied, and the second is focused on Southeast Asia, such as #armenia, #abudhabi, or #masdarcity.

### Bill Gates

The data from the Bill Gates conversation are similar to those obtained in the case of hydroxychloroquine. Trump-related hashtags were very common (#kag, #maga, and #qanon) in both bots and nonbots. The centrality and degree values are among the highest, as can be seen in Table 2. There were also new hashtags related to this type of political movement that only appears in this conversation, such as #crimesagainsthumanity, #gatesofhell, or #greatawakening. In addition, hashtags disparaging the figure of Bill Gates are also common, such as #saynotobillgates or #billgatesisevil.

We identified 5 communities of hashtags (Multimedia Appendix 1). Among the 3 largest communities, the number of bots is higher than the number of nonbots in the second one. In this community, the most frequent hashtags are #trump, #depopulationagenda, #eugenetics, #repubicans, #auspol, #qanon, and #americafirst. The hashtags, as mentioned above, are related to Trump or against some figures who have publicly supported vaccines. Examples are #trump, #americafirst, or #faucifraud. These hashtags can also be found in the first community, where the percentage of both account types is similar. However, in this community, the number of bots is not higher than that of nonbots. In the third community, the number of nonbots is higher than bots. Most hashtags in this community mention COVID-19 (#coronaviruschallenge, #coronavirusbill, #coronaviruschina, and #coronavirusnewyork), but other hashtags such as #hoaxvirus, #tedconnnect, #freedomovefear, or #trumpisevil also appear.

### 5G

Regarding 5G, hashtags related to technology or news were the predominant ones in the case of nonbots, such as #techwar-#tradewar or #bbcaq-#itvnews. On the other hand, in the case of bots, the hashtags continue to mention geographical areas: #america-#china and #america-#lka. There are other hashtags with higher intensity, for example, #chinesecoronavirus-#democratshateamerica or #conspiracytheories-#technology. As can be seen in Table 2, the #china hashtag gets the highest PageRank value, followed by #pandemic and #wuhan. In addition, #china has 42 degrees of connectivity, doubling the value of the second, which is #pandemic with 27 connections. But above all, these values indicate the central place these hashtags take in the conversation. On the one hand, the high degree indicates they co-occur with many different hashtags. On the other hand, a high betweenness value indicates a central place in the network.

This time, the algorithm found 5 different communities of hashtags (Multimedia Appendix 1). The presence of bots is higher than nonbots in the first 3. The first is related to #tech, #bigdata, #cibersecurity, and so on. The second one is focused on #conspiracytheories, #digitalskynet, and #misinformation. The third is focused on China, with hashtags such as #batflu, #chinesevirus, and #huaweithis. The last 2 communities, where the level of nonbots is higher, are formed by varied hashtags. The fourth community is formed by hashtags such as #kag or #maga. The fifth one contains hashtags mentioning rumors or disinformation: #fakenews, #disinformation, and #democrathoax. In this community, it is worth mentioning the appearance of hashtags related to #blacklivesmatter, such as #racism, #blacklivesmatteraustralia, or #policebrutality.

### UV

In this case, the appearance of technology-related hashtags (#ai and #healthtech) is even more noticeable, especially in the case of bots (Table 2). On the other hand, the most common hashtags are #batflu-#quarantine in the case of nonbots. Concerning the 6 communities we found (Multimedia Appendix 1), in the first 3, the number of nonbots is higher. The subject matter of these communities is related to politicians (#trump, #joebiden, and #berniesanders), technology (#artificialintelligence, #bioinformatics, and #machinelearning), or more specifically to technological innovation (#health, #innovation, #coronavirusnewyorkty, and #smartcities).

## Discussion

### Principal Findings

This study examined the use of hashtags by social bots on Twitter during the early stages of the COVID-19 pandemic. By analyzing the co-occurrence of hashtags, we were able to identify differences between accounts classified as bots and nonbots. We used Louvain communities to further classify these co-occurrences and found consistent differences in hashtag usage between the 2 groups. We used social network analysis based on the co-occurrence of hashtags to take advantage of hashtags as key elements of online texts and understand how different users tag posts.

The analysis of hashtags provided several key insights into attitudes toward the COVID-19 pandemic and related behaviors. We consistently observed differences between bots and nonbots. In the case of bots, it was more common to find co-occurrences of hashtags related to political movements, particularly those on the right wing and related to Trump. This is consistent with findings in the literature showing a higher presence of conservatives in topics related to misinformation about COVID-19 pandemic [45].

In the conversation about vaccines, we observed that bots used hashtags related to fake news, such as #billgates and #china, more frequently. This analysis also identified specific uninformative hashtags (#ccpvirus and #chinesevirus) associated with anti-Asian sentiment [18]. Other hashtags expressed different opinions, such as criticism (#billgateisevil) or hate (#chinaliedpeopledied). It is worth noting that most of the tweets posted by nonbot users came from official accounts of institutions such as the World Health Organization, ministries of health, or entities related to public health. These messages focused on reporting on the evolution of the pandemic; the number of deaths; infection rates; and the health measures implemented, such as lockdowns and vaccination campaigns to contain the spread of the virus.

In our analysis of the conversation related to hydroxychloroquine, we identified 2 distinct communities of hashtags. One group was related to public health or medicine, while the other group was related to political movements and associated with Trump. Other studies have also found that Trump was involved in this conversation [46,47]. However, we also found that some of the hashtags in the conversation about hydroxychloroquine related to scientific facts. These differences suggest a highly polarized conversation with scientific arguments pitted against controversial political campaigns.

According to one of these studies [47], accounts with a higher impact on topics related to hydroxychloroquine disinformation were more likely to support President Trump. In addition, these types of content had a higher volume of tweets, longer duration in time, and greater echo. Our findings on the number of bots in these communities with politicized hashtags would partly explain the permanence over time and high echo values. Bots amplify these debates and increase the impact of the messages they disseminate [29,48,49]. However, our results also identify communities with anti–President Trump hashtags and higher numbers of bots. Liberals also engage in these conversations, although to a lesser extent than Conservatives [45].

These findings are extensible to topics such as the military or Bill Gates, where the conversation has been highly politicized and permeated with fake news. According to the results obtained, Trump occupied a leading role in the Twitter conversations during the period analyzed. This fact has also been noted in other previous works. Trump publicly supported the use of hydroxychloroquine and other drugs to combat the advance of the COVID-19 pandemic, with its corresponding impact on increased searches [50]. In addition, Bill Gates is often the protagonist in conspiracy theories [51].

### Limitations and Strengths

There are several factors to consider when categorizing accounts as nonbot or bot. Botometer is backed by a large volume of research, but its effectiveness has been debated. It is important to remember that Botometer only provides a probability that an account is a bot, not a definitive classification. To get the most accurate results, it is recommended to compare probability distribution. However, in some cases it may be necessary to establish a binary classification for research purposes. In such cases, previous research has shown that using a cutoff value and comparing the results is a successful strategy [52].

It is important to consider the language constraint of this study. Only selecting tweets written in English may limit the focus to actors and events from English-speaking countries. In addition, no geographic limitations were placed on the collection of tweets, which allows for a larger volume of data but may also make it difficult to interpret results. It is also worth noting that the tweets analyzed in this study were from the early stages of the pandemic, and conversations and topics may have evolved over time.

### Conclusion

Our analysis of hashtag usage on Twitter showed that there were differences in the patterns of use between bot and nonbot accounts. By grouping hashtags based on co-occurrence, we were able to identify distinct patterns in the usage of hashtags. On controversial or highly polarized issues, the hashtags used often pertained to the campaign or movement being promoted, with a significant portion related to Trump. In some cases, hashtags opposing these movements were also identified. On less polarized topics, hashtag usage was more diverse and included references to specific geographic locations or social groups. This analysis method can be useful in detecting hashtags that may be linked to fake news or misinformation, or in tracing the spread of such content on social media platforms.

## Appendix

Multimedia Appendix 1 Bot distribution by topic.

## Abbreviations

API: application programming interface
NLP: natural language processing
SNA: social network analysis
