# Supplementary figures and images for: Unraveling the Use of Disinformation Hashtags by Social Bots During the COVID-19 Pandemic: Social Networks Analysis
Source: JMIR Infodemiology. 2025 Jan 9;5:e50021. doi: 10.2196/50021 (PMC11757974; doi:10.2196/50021)

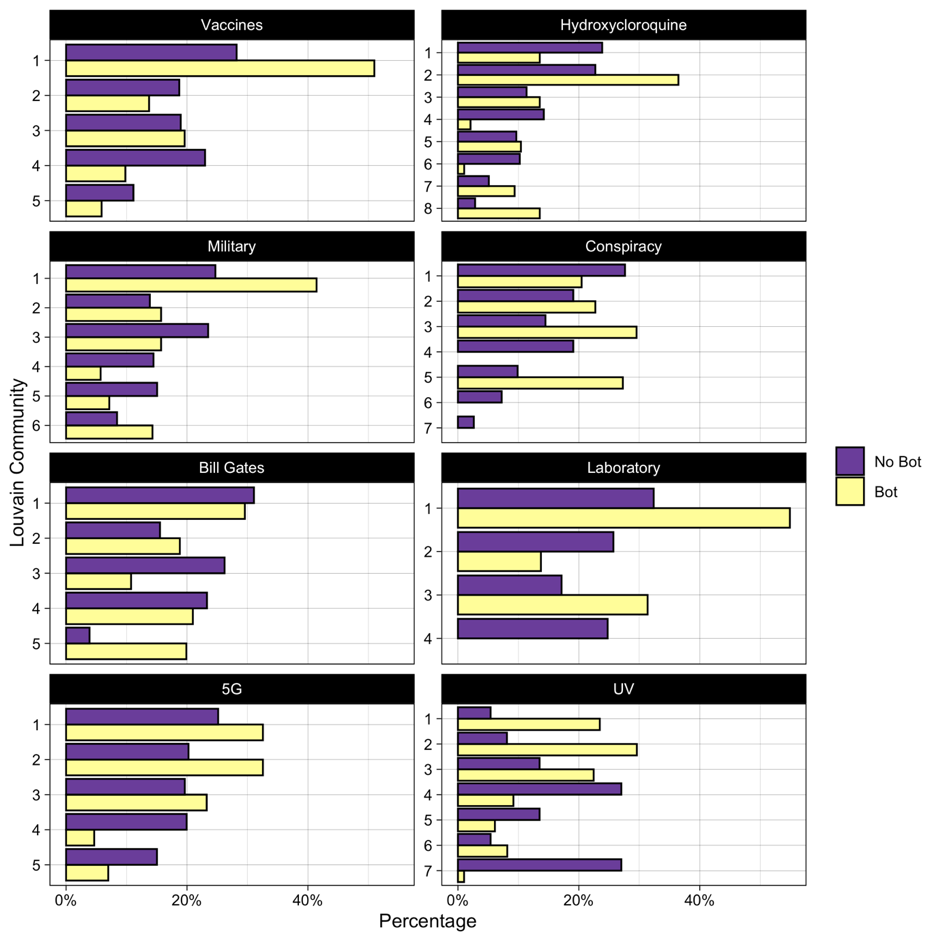

Supplement: Multimedia Appendix 1 [file infodemiology_v5i1e50021_app1.png]
